# Owner reported diseases of working equids in central Ethiopia

**DOI:** 10.1111/evj.12633

**Published:** 2016-10-13

**Authors:** A. P. Stringer, R. M. Christley, C. E. Bell, F. Gebreab, G. Tefera, K. Reed, A. Trawford, G. L. Pinchbeck

**Affiliations:** ^1^ Institute of Infection and Global HealthUniversity of LiverpoolLiverpoolUK; ^2^ Royal (Dick) School of Veterinary StudiesUniversity of Edinburgh, RoslinMidlothianUK; ^3^ College of Veterinary Medicine and AgricultureAddis Ababa UniversityDebre ZeitEthiopia; ^4^ SPANALondonUK; ^5^ The Donkey SanctuarySidmouth, DevonUK

**Keywords:** horse, working equid, participatory, disease, health, donkey

## Abstract

**Reasons for performing study:**

Working horses, donkeys and mules suffer from numerous diseases and clinical problems. However, there is little information on what owners perceive as important health concerns in their working animals.

**Objectives:**

To identify and prioritise with owners the diseases and other health concerns in working equids in central Ethiopia using participatory methodologies.

**Study design:**

Participatory situation analysis (PSA).

**Methods:**

The study was conducted with carthorse‐ and donkey‐owners in 16 sites in central Ethiopia. Multiple participatory methodologies were utilised, including ranking, matrices and focus group discussions. Owners’ perceptions on frequency, importance, morbidity and mortality of volunteered diseases and the clinical signs that owners attributed to each disease were obtained; information regarding the impact of these diseases and health concerns was also sought.

**Results:**

A total of 40 separate disease and health problems were volunteered by carthorse‐ and donkey‐owners. Horse‐owners volunteered a musculoskeletal syndrome (with the local name ‘bird’, clinical signs suggest possible disease pathologies including equine exertional rhabdomyolysis), colic and epizootic lymphangitis most frequently, whereas donkey‐owners volunteered sarcoids, nasal discharge and wounds to occur most frequently. One problem (coughing) was volunteered frequently by both horse‐ and donkey‐owners. Owners demonstrated knowledge of differing manifestations and severity of these problems, which resulted in differing impacts on the working ability of the animal.

**Conclusions:**

Although many of the diseases and clinical signs had been previously reported, this study also identified some previously unreported priorities such as rabies in donkeys, an unidentified musculoskeletal syndrome in horses and respiratory signs in both horses and donkeys. The information gathered during this participatory study with owners may be used to inform future veterinary and educational programme interventions, as well as identify future research priorities.

## Introduction

It is estimated that the total world equine population is around 112 million, although this is very likely to be a gross underestimate 1. It is reported that two million horses, 0.36 million mules and seven million donkeys comprise the working equid population in Ethiopia and this represents the largest population of donkeys in Africa 2. Working equids have a direct impact on the lives of rural people by reducing the transport burdens of water, fuel, wood and goods 3, transporting people and in some areas for agricultural purposes 3, 4, 5.

Disease and health problems affecting working equids and their productivity in Ethiopia have been previously documented and include harness‐related wounds and sores 6, 7, 8, 9, 10, colic 7, 8, coughing and nasal discharge 7, 8, 11, epizootic lymphangitis (EZL) 12, 13, 14, 15, 16, African horse sickness 17, 18, 19, helminthiasis 10, 20, 21, 22, ocular disease 23, lameness 7, 24 and sarcoids 10, 25. Many of these health and welfare problems are also found in other low‐ and middle‐income countries 26, 27, 28, 29, 30, 31, 32.

There is, however, little information on working equids‐owners’ perceptions and prioritisation of these disease and health problems in Ethiopia, or elsewhere. In two participatory studies in Ethiopia, the most frequently encountered problems were respiratory problems, colic, musculoskeletal and hoof problems, back sores and EZL 33, 34. Participatory methods are frequently used in livestock research and are becoming more frequently used in studies on working equid health. The aim of this study was to identify and prioritise the disease and health concerns that affect working equids according to their owners using participatory approaches. Owners’ perceptions on frequency, importance, morbidity and mortality of volunteered diseases and the clinical signs that owners attributed to each disease were also obtained, enabling the targeting of programmatic interventions and future research.

## Materials and methods

### Survey sites

The participatory situation analysis (PSA) was conducted between January and March 2008. Sites were selected from three regions in central Ethiopia: Oromia, Amhara and Southern Nations, Nationalities and People's Region (SNNPR) (Supplementary Items 1 and 2). A total of 16 sites from 12 different towns and villages were selected in a range of agroecological zones and comprised either rural villages that contained predominately donkey‐owners, or towns with predominantly horse‐owners (Supplementary Items 3 and 4). Sites varied between those located in the highland regions, (e.g. Debre Brehan, 2840 m) to those located in the lowland regions (e.g. Ziway, 1643 m). Towns and villages were selected based on the logistics of transportation, but were chosen to be representative of other towns and villages in the region. Sites were designated as either ‘exposed’ if they had previous known exposure to an equine nongovernmental organisation (NGO) or equine education/research programme, or ‘unexposed’ if they were a naive population with regards to the above criteria.

### Survey participants

Participants were eligible for inclusion in the PSA if they lived within the selected town or village and owned, or used, a donkey or horse. Donkeys in these sites were primarily used to carry water, firewood and agricultural products, whereas horses were primarily used to carry people (and goods) in carts. Participation was voluntary with no financial incentive offered. Participants were selected using two different methods. In exposed towns and villages, owners were approached during their attendance at the mobile veterinary clinic. Participants from unexposed towns and villages were recruited using development agents assigned from the relevant Bureau of Agriculture. Participants were asked to gather at a designated site at a specific time.

### Participatory situation analysis

The PSA was conducted in either of two regional languages (Amharic and Afan Oromo) as dictated by the participants’ preference. One of the authors (G.T.; an Ethiopian national) is fluent in Amharic, Afan Oromo and English and was the primary facilitator and translator for the PSA. He had previous experience in the field of animal health and had received training in participatory approaches. The PSA was based on five questions in a semi‐structured interview (SSI) format and utilised a number of different methodologies (Supplementary Item 5). The PSA was piloted with horse‐and donkey‐owners from other villages and towns not involved in this study and questions underwent reverse translation (the process of translating study questions back into English from local Ethiopian languages to check for accuracy in original translation) prior to commencing the study.

Participants were initially asked to volunteer all disease and health problems that afflicted either their donkeys or horses. Following this, the group was asked to arrive at a consensus on a ranking of these diseases in the order of how commonly they were encountered. For each of the volunteered diseases, the group was asked to form a consensus on how long an animal would be out of work and unable to perform its duties if afflicted with a particular disease. In open discussion, owners were then asked to describe the clinical signs associated with the volunteered problems. Piloting on 10 groups in four sites led to minor modifications to the interview questions. These modifications were made to ensure that an accurate translation of the researchers intended questions was obtained.

### Data recording and analysis

Data were initially translated and recorded in written format by a dedicated recorder in English, and using digital photos of completed matrix boards. These data were then entered into a Microsoft Excel 7 spreadsheet programme^1^. The R language for statistical computing^2^ was used for analysis of quantitative data and for plotting figures and NVIVO 8^3^ was used to aid thematic coding of data obtained during the open discussions. Owners volunteered disease and health problems using local names in either of the two regional dialects (Afan Oromo and Amharic). These local names were translated into equivalent English names during the analytical stage; where locally named conditions were not directly translatable the clinical signs described by the participants were compared with standard descriptions of diseases in veterinary textbooks used to assist identification of the disease or health problem. Health problems were ranked in two ways: first simply by the number of times individual groups volunteered the specific problem (count rank) and second using a weighted score depending on where groups ranked each problem (score rank), in which the problem ranked first was given a score of ten, nine for second place, continuing until all problems were assigned a score. We calculated these ranks for unexposed and exposed sites and both sites combined.

## Results

A total of 160 participants (two groups of five participants in each of 16 sites) participated in this study. Forty separate disease and health problems were volunteered by horse‐ and donkey‐owners (Supplementary Item 6). Data saturation was achieved during the group discussions. As the number of group discussions progressed, no new disease entities were volunteered. Only one condition (coughing) was volunteered frequently by both horse‐ and donkey‐owners (Tables 1 and 2). Numerous problems were volunteered more frequently by horse‐owners (e.g. musculoskeletal syndrome with the local name ‘bird’, clinical signs suggest possible disease pathologies including equine exertional rhabdomyolysis, EZL and colic) or donkey‐owners (e.g. sarcoids, nasal discharge and wounds). Donkey owners perceived wounds to be the most common problem affecting their animals in unexposed towns, followed by sarcoids, nasal discharge and coughing (Table 1). In exposed towns, donkey‐owners perceived nasal discharge to be the most common problem followed by coughing (Table 1). Horse‐owners perceived ‘bird’ to be the most common problem affecting their animals in unexposed sites, followed by EZL and colic (Table 2). In exposed sites, EZL was also the most common problem followed by coughing, colic and ‘bird’ (Table 2). In the descriptions of the health problems provided, owners differentiated between ‘lip wounds’ and ‘mouth wounds’. For ‘lip wounds’, owners described a ‘small swelling inside upper lip’ and ‘wound on upper lip’, whereas for mouth wounds, owners described a health problem which caused ‘gum down over teeth’. This second health issue (mouth wound/gum down) is consistent with a health problem documented in another published study 26. Owners volunteered a health problem they named as either ‘dog disease’ or rabies and were able to provide descriptions of donkeys with clinical signs consistent with rabies (i.e. change of behaviour, vocalisation, aggression towards other animals/man, self‐traumatising behaviour). The results across towns and villages (taking into account differences between exposed and unexposed sites) were generally similar for each species; however, there were some differences at high altitude (Debre Brehan) where horse owners did not volunteer EZL as a health problem.

**Table 1:** Top 10 ranked (using combined count rank) health problems volunteered by 16 groups of donkey‐owners at eight different sites in Central Ethiopia

Health problem	Unexposed sites		Exposed sites		Combined	
	Count rank	Score rank	Count rank	Score rank	Count rank	Score rank
Nasal discharge	3=	3=	1	1	1	1
Coughing	3=	3=	2	2	2	2
Sarcoids	1=	2	3=	5	3	4
Wound	1=	1	7	7	4	3
Colic	6	6	8=	–	5=	9
Bloating	9=	10=	3=	3	5=	5
Rabies	–	–	3=	4	5=	6
Parasites^a^	9=	9	3=	6	5=	7
Day disease^b^	7=	7=	8=	8	9=	8
No urination	5	5	–	–	9=	10
Weight loss	–	10=	8=	9	11=	–
Hoof problem	–	–	8=	10	11=	–
Musculoskeletal^c^	7=	7=	–	–	–	–

Count rank, number of times individual groups volunteered the specific problem.

Score rank, a score weighted depending on where groups ranked each problem (problem ranked first given a score of ten, nine for second place, continuing until all problems were assigned a score).

a Parasites, internal parasites (described by owners as worms).

b Day disease, unknown disease.

c Musculoskeletal, unknown musculoskeletal syndrome.

**Table 2:** Top 10 ranked (using combined count rank) health problems volunteered by 16 groups of horse‐owners at eight different sites in Central Ethiopia

Health problem	Unexposed sites		Exposed sites		Combined	
	Count rank	Score rank	Count rank	Score rank	Count rank	Score rank
EZL	1=	2	1=	1	1	1
Colic	3	3	3	3	2	2=
Musculoskeletal^a^	1=	1	4=	4	3=	2=
Coughing	5=	9	1=	2	3=	4
Lip wound	5=	10	4=	6=	5	5
Parasites^b^	–	–	4=	5	6=	6
Foot abscess	–	–	4=	6=	6=	7
Swollen legs	5=	4	9=	13	8=	8
Nasal discharge	5=	5	9=	12	8=	9=
No urination	–	11=	9=	8	10=	9=
Mouth lesion	5=	7	–	–	10=	11
Day disease^c^	4	–	–	9	10=	12
Wound	–	–	8	10	10=	13
Anthrax	5=	6	–	–	–	–
Swelling above eye	5=	7	–	–	–	–
Joint swelling	–	11=	–	–	–	–
Bloating	–	–	9=	11	–	–

Count rank, number of times individual groups volunteered the specific problem.

Score rank, a score weighted depending on where groups ranked each problem (problem ranked first given a score of ten, nine for second place, continuing until all problems were assigned a score).

a Parasites, internal parasites (described by owners as worms).

b Musculoskeletal, musculoskeletal syndrome (with the local name ‘bird’, clinical signs suggest possible disease pathologies including equine exertional rhabdomyolysis).

c Day disease, unknown disease causing death within one day (clinical signs suggest possible aetiologies, particularly African Horses Sickness virus).

EZL, epizootic lymphangitis.

**Figure 1:**
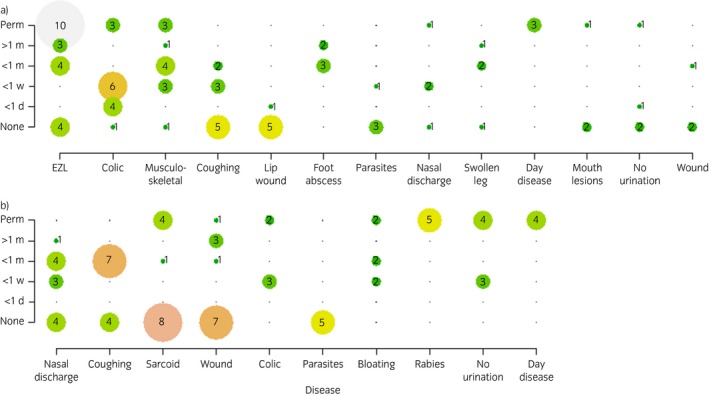
Impact on working ability of the most frequently volunteered diseases of a) horses and b) donkeys in Central Ethiopia. The size and colour of the circle in each graph represent the number of times this category was volunteered, also shown by the number in the circle. Key (x axis): <1 d, <1 day; <1 w, <1 week; <1 m, <1 month; >1 m, >1 month; perm, permanently out of work; EZL, epizootic lymphangitis

Horse‐ and donkey‐owners identified the impact that each problem had on the ability of that equid to perform its role and to work and these were recorded in matrices. Owners demonstrated knowledge of differing manifestations and severity of volunteered problems, resulting in a differing amount of time off work (Fig 1). Groups could volunteer more than one answer for each volunteered problem. For example, horse‐owners reported two distinct severities of EZL (Fig 1a); one manifestation that does not affect the horse's ability to work and another that leads to significant time off work and ultimately to the horse not being able to work. The same distribution is seen with colic in horses, with the majority of cases causing the horse to be off work for less than a week, but a small number of cases leading to the horse being permanently out of work or death. However, coughing and lip wounds both show that the majority of these cases have little impact on the ability of horses to work. For donkey‐owners (Fig 1b), nasal discharge shows two distinct manifestations of the disease: some donkeys remain working whilst others required 1 week to a month off work. A similar distribution was seen with coughing. In the majority of groups, owners indicated that donkeys with sarcoids, wounds and parasites were not deemed to require any time off work.

Both donkey‐ and horse‐owners were able to attribute various clinical signs to the volunteered problems (Supplementary Items 7 and 8). Thematic coding revealed that donkey‐owners had knowledge of the location of wounds on their donkeys and knowledge of common sites for sarcoids. Coding revealed that the thematic descriptions given by donkey‐owners to colic included bloating, anuria and anorexia. These are all clinical signs that may be consistent with a donkey affected by colic, a frequently reported problem in working donkeys 8. Horse‐owners had considerable knowledge of the clinical signs frequently observed in a horse affected by colic which included rolling and restlessness. For EZL, the most commonly volunteered problem amongst horses, owners accurately described the clinical appearance of the disease and the most commonly reported locations of lesions. For the musculoskeletal syndrome, owners reported that the two most frequently observed signs were a change in musculoskeletal system anatomy (e.g. muscle tone or limb deformity) and a change in locomotion.

## Discussion

Using participatory methodologies we were able to ascertain an accurate picture of the knowledge and perceptions of Ethiopian working equid‐owners concerning the major disease and health concerns of working horses and donkeys. In our study, 40 separate disease, clinical signs and health problems were described by horse‐ and donkey‐owners, which highlights both the diversity of diseases that afflict these animals and the awareness of owners to these different disease problems.

The problems that donkey‐owners perceived to be the most common were triangulated with other sources of information on diseases of working donkeys in Ethiopia and there was strong agreement. Triangulation is the use of two or more methods, data sources, observers or investigators, or theories within the same study 35. For example, clinical treatment records from one NGO (The Donkey Sanctuary, unpublished data) showed that wounds, back sores and respiratory problems (coughing and nasal discharge) were the three most commonly presented conditions at their mobile veterinary clinics, whilst one survey of 200 Ethiopian donkey owners found the most prevalent health problems encountered was coughing and nasal discharge 8, followed by wounds and colic. Donkey‐owners in another study ranked respiratory disease as the disease problem most afflicting their donkeys 36, whilst focus group discussions in a different study identified respiratory problems (coughing and nasal discharge), colic and back sores as the health problems most commonly affecting equids 33. The difference in the ranking of wounds on donkeys between the exposed and unexposed sites could potentially be explained by the successful impact of NGO interventions (in exposed sites) aimed at reducing wounds through harness modification and owner management changes.

Owners in this study ranked rabies in the top five diseases to affect their donkeys and yet this disease is not commonly seen in cases presenting at NGO clinics. Reasons for this could include clinical cases dying before presenting at clinic or owners recognising clinical signs and abandoning animals or performing euthanasia. Owners in this study had a detailed knowledge of rabies, its transmission and the disease prognosis. Rabies has been identified in a number of species in Ethiopia including working donkeys 36, 37, 38. This finding concerning owners’ perception of rabies in their donkeys is of particular importance due to its zoonotic potential and lack of current veterinary interventions to reduce rabies within the equid population.

When the problems that horse‐owners perceived to be the most common were triangulated with the clinical records of another NGO (Society for the Protection of Animals Abroad, unpublished data), these conditions were in the top five disease problems most commonly presented to veterinary clinics for treatment. There is also agreement between this study's findings and those from other participatory studies 33, 34 and other studies 7. One participatory study identified EZL, hoof problems, ‘bird’, colic and respiratory problems as the top five disease problems affecting working carthorses 34, whilst another study identified respiratory problems (coughing and nasal discharge), colic, back sores and EZL as the health problems most frequently identified by working equid‐owners 33. Whilst a number of health problems were volunteered by both unexposed and exposed groups (including coughing, colic and lip wounds), horse‐owners in exposed groups more frequently volunteered coughing, lip wounds and parasites than unexposed groups. One potential explanation for this finding could be that intervention programmes organised by NGOs have raised awareness amongst owners in exposed sites to these problems compared with owners in unexposed sites.

Epizootic lymphangitis has been well documented as a major problem of horses in Ethiopia that has a major impact on the socioeconomics of owners 12, 15, 16, 39. Studies in other developing countries have shown lameness, gait abnormalities and foot problems to be extremely common in all working equids 28, 32. In one study, 74% of 201 interviewed Ethiopian carthorse‐owners said lameness was a disease constraint, ranking it fourth out of all diseases volunteered 7. In this study, owners did differentiate between a volunteered musculoskeletal syndrome (whose local name was ‘bird’) and foot abscesses, which they also volunteered as a top ten problem in this study. It was not possible to positively attribute a specific disease to this syndrome (‘bird’), although the clinical signs suggest possible disease pathologies including equine exertional rhabdomyolysis (owners reported that the two most frequently observed signs were a change in musculoskeletal system anatomy [e.g. muscle tone] and a change in locomotion), equine exertional rhabdomyolysis has been reported in working equids in other countries 40. Further research is required to explore this health problem in greater detail with owners, preferably involving clinical examinations and diagnostic investigation of the suspected equids with this syndrome.

Harness‐related wounds have previously been documented as one of the most prevalent health problems in equids in Ethiopia 6, 7, 8 and this is consistent with literature from other developing countries 29, 30, 31. However, in this study, owners perceived wounds on horses to be less of a problem than was expected by the authors, suggesting that although they may be prevalent as previously reported by published literature 6, they may be perceived as lesser significance to horse‐owners in this study, or they are not as common in this population due to NGO programmatic interventions.

Both carthorse‐ and donkey‐owners were able to identify differing clinical manifestations or severities of diseases that had varying degrees of impact on the animal's ability to work. Horse‐owners’ descriptions of the presenting clinical signs of EZL were consistent with the clinical pattern seen in an EZL case, with progression from a mild case to a more severe case that is not treatable and ultimately leads to horses being abandoned 41. A similar distribution for colic is also consistent with the clinical pattern seen in colic cases, with the majority of clinical cases resolving in a short period, whilst a minority are catastrophic abdominal events leading to death 42. Donkey‐owners described the impact of nasal discharge as one of two patterns, either no impact, or that of requiring 1 week to a month off work. This may be due to the different possible aetiologies causing nasal discharge and coughing in donkeys. Some clinical cases could be mild, whilst others such as Strangles (*Streptococcus equi* infection) have the potential to be more severe. Little is known about the aetiology of respiratory disease in working horses and donkeys. In one participatory study, 44 different respiratory syndromes were recognised by horse‐owners 11. A subsequent cross‐sectional study across 19 sites suggested exposure to *Streptococcus equi* subsp. *equi* was approximately 13%, equine viral arteritis 3.4% and exposure to equine influenza rare 11. While both studies supported infectious diseases as one aetiology for respiratory disease, other possible aetiologies could include environmental and management factors. Further research is needed to explore the other potential causes of respiratory disease.

This study relied on participants volunteering information about clinical signs and diseases and we were unable to validate the accuracy of their identification. For example, other neurological signs may present as rabies and hence may be misclassified and it may be difficult to diagnose some diseases such as anthrax without laboratory confirmation. It is also possible that due to language translation issues we may have misclassified some diseases, a potential limitation of the study. The participatory approaches used in this study to gather predominately qualitative information have their strengths and weaknesses. The strengths of participatory approaches are its ability to generate locally specific information that has local validity 35 and allows in‐depth discussions that provide a better opportunity for understanding owners’ concerns and perceptions 26. Participatory epidemiology has a role to play in strengthening stakeholder involvement in the analysis of disease problems by ensuring that both veterinarians and researchers understand the perspectives and priorities of working equid‐owners and helping ensure community ownership of research findings 42, 43. This is of importance as prioritisation of an intervention to address problems perceived as important only by external parties is unlikely to be understood or valued by the targeted owners in the absence of engagement with owners to highlight those problems and their impact on both animal and owner 44.

Potential weaknesses of participatory approaches are their specificity for the geographical area in which the study is conducted and a lack of direct objective measurement of disease and health problems that can be obtained with classical quantitative epidemiological studies, such as a cross‐sectional survey. The locations and the owners who participated in this study were deemed to be representative of other locations and working equid‐owners in the region. However, there is potential for selection bias as no random sampling process was utilised during this study to select either locations or participants and owners who volunteered to participate in this study may differ in some way from those who did not want to participate. Both these issues could potentially lead to the locations and participants being a nonrepresentative sample of the intended target population.

This study has identified numerous disease and health constraints that impact on the working ability of equids. It has also highlighted differences in what owners perceive as common and important diseases that affect their equids and what service providers most commonly treat. The results presented here have been used to inform the design and content of educational interventions that aimed to increase the knowledge of working equid‐owners about the health and welfare of their animals 45, identify areas requiring further research 11 and highlight diseases that owners perceive as important requiring preventive interventions. Whilst it should be recognising that infectious diseases are only one of several key welfare challenges faced by working equids, developing improved diagnostics (for EZL) and improved disease surveillance (for different AHS serotypes) would aid in the prevention and control of these diseases. In addition, further research should focus on impact assessment of interventions on both animals and owners 26.

## Conclusion

The participatory approaches utilised in this study allowed rapid identification and prioritisation of major disease and health concerns of working equid‐owners in Ethiopia. This study compliments previous studies by focusing on owners’ knowledge and perceptions of working equid health and disease problems. The information gathered during this PSA may be used to inform decisions regarding the targeting of educational interventions and clinical programmes and is of benefit to veterinarians, government and NGOs in identifying areas requiring further research. This study also identified that certain diseases remain poorly characterised by owners and potentially need definitive identification before effective interventions can be developed. It is recommended that both quantitative and qualitative approaches are utilised as part of a comprehensive needs assessment prior to defining and prioritising future interventions.

## Author's declaration of interests

The authors have declared no competing interests.

## Ethical animal research

The study was conducted in accordance with the research ethics requirements of the Faculty of Veterinary Science at the University of Liverpool. Informed verbal consent was obtained from all participants involved in the study following a short introductory briefing concerning the purpose of the PSA. This briefing also stressed that participation in the study was voluntary and that people were free to leave at any time.

## Sources of funding

The authors are grateful to the Wellcome Trust Livestock for Life grant awarded to G.L. Pinchbeck for the funding of this project.

## Authorship

A.P. Stringer contributed to study design, study execution, data analysis and interpretation and preparation of the manuscript. R.M. Christley, C.E. Bell and G.L. Pinchbeck contributed to study design, data analysis and interpretation and preparation of the manuscript. F. Gebreab, K. Reed and A. Trawford contributed to study design. G. Tefera contributed to study design, study execution and data analysis and interpretation. All authors gave their final approval of the manuscript.

## Supporting information


**Supplementary Item 1.** Map of Ethiopia showing administrative regions.

**Supplementary Item 2.** Map showing study locations in Ethiopia.

**Supplementary Item 3.** Information on the towns and villages selected in the participatory situation analysis (PSA).

**Supplementary Item 4.** Data on the population of horses and donkeys in each of the study regions.

**Supplementary Item 5.** Semi‐structured interview questions and participatory methodologies used in the PSA.

**Supplementary Item 6.** Complete list of all disease and health problems volunteered by 32 groups of horse‐ and donkey‐owners at 16 sites in Central Ethiopia.

**Supplementary Item 7.** Thematically coded clinical signs attributed by donkey‐owners to volunteered disease problems.

**Supplementary Item 8.** Thematically coded clinical signs attributed by horse‐owners to volunteered disease problems.

## Manufacturers' addresses

1 Microsoft Corporation, Redmond, Washington, USA.

2
http://www.r-project.org

3 QSR International (Americas) Inc., Burlington, Massachusetts, USA.
