# Supplementary material for: Owner reported diseases of working equids in central Ethiopia
Source: Equine Vet J. 2016 Oct 13;49(4):501–6. doi: 10.1111/evj.12633 (PMC5484383; doi:10.1111/evj.12633)

Equine Veterinary Journal

Supplementary Item 1: Map of Ethiopia showing administrative regions (Source: https://en.wikipedia.org/wiki/Subdivisions_of_Ethiopia).

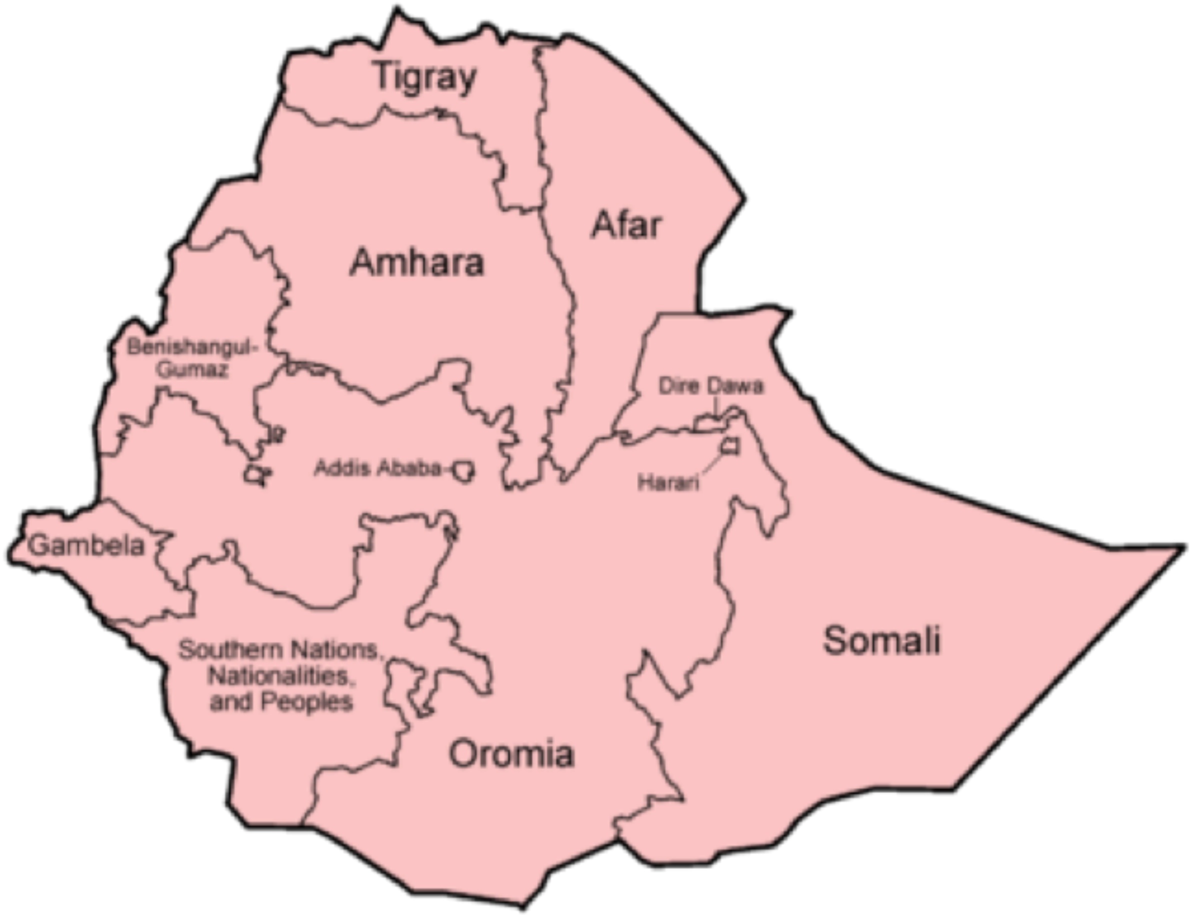

Supplement: Supplementary file 1 — Supplementary Item 1. Map of Ethiopia showing administrative regions. [file EVJ-49-501-s001.pdf]
