# Supplementary figures and images for: Owner reported diseases of working equids in central Ethiopia
Source: Equine Vet J. 2016 Oct 13;49(4):501–6. doi: 10.1111/evj.12633 (PMC5484383; doi:10.1111/evj.12633)

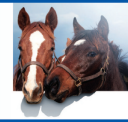

**Supplementary Item 2:** Map showing study locations in Ethiopia.

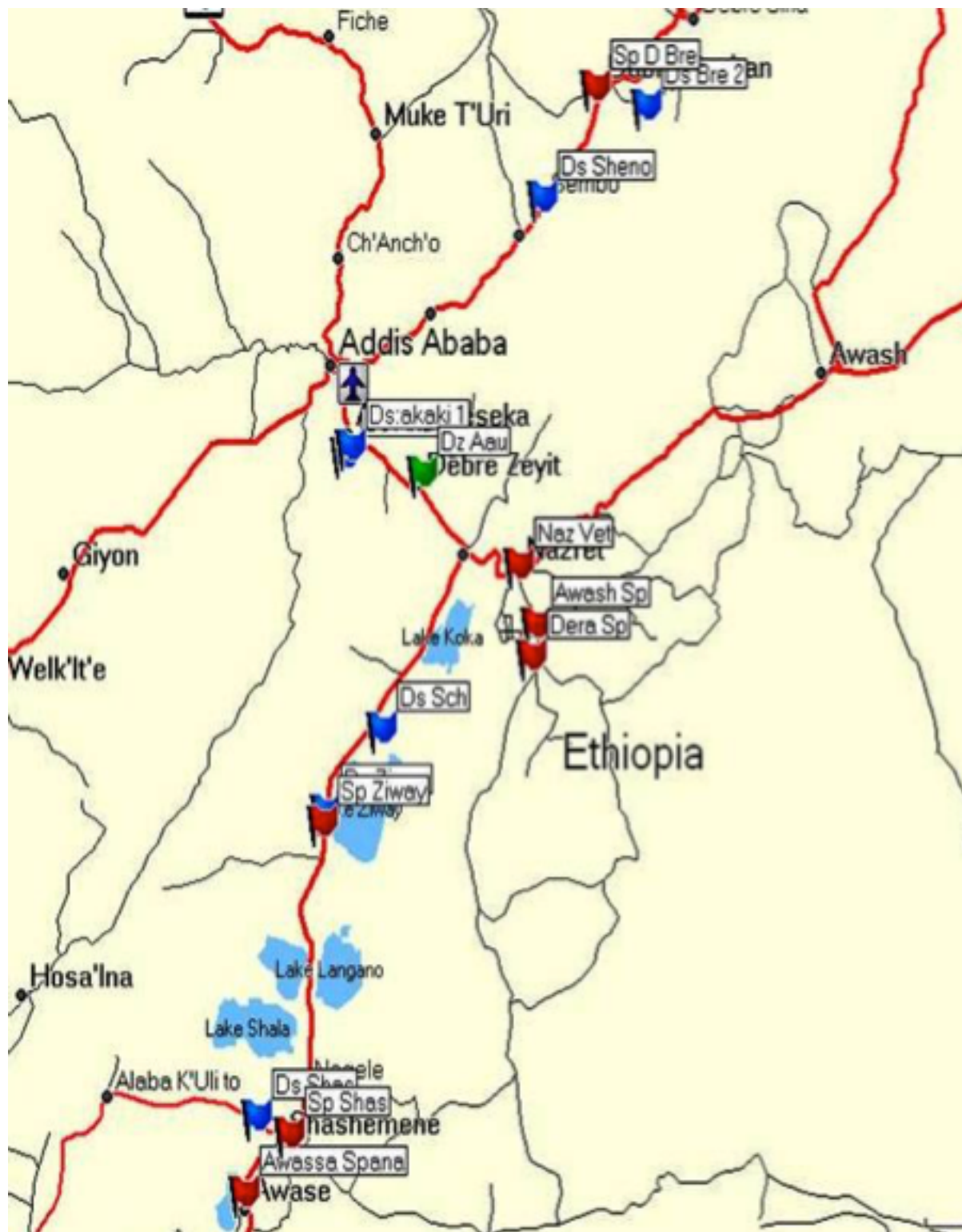

Supplement: Supplementary file 2 — Supplementary Item 2. Map showing study locations in Ethiopia. [file EVJ-49-501-s002.pdf]
