# Supplementary material for: Owner reported diseases of working equids in central Ethiopia
Source: Equine Vet J. 2016 Oct 13;49(4):501–6. doi: 10.1111/evj.12633 (PMC5484383; doi:10.1111/evj.12633)
Supplement: Supplementary file 3 — Supplementary Item 3. Information on the towns and villages selected in the participatory situation analysis (PSA). [file EVJ-49-501-s003.pdf]

Equine Veterinary Journal

Supplementary Item 3: Information on the towns and villages selected in the Participatory Situation Analysis (PSA).

Town/Village Name	***Horse owners***	***Donkey owners***	***Region***	***Zone***	***Woreda (Municipality)***
Awash	U		Oromia	East Shewa	Adama
Awassa	E		SNNPR	Sidama	Awassa
Debre Brehan	E	E	Amhara	North Shewa	Basuna Warano
Debre Zeyit	E	E	Oromia	East Shewa	Ada
Dera	U		Oromia	Arsi	Dodota
Gamo		E	Oromia	East Shewa	Dugda
Gemeda (Akaki)		E	Oromia	Addis Ababa Area	Akaki
Merino (Akaki)		E	Oromia	Addis Ababa Area	Akaki
Nazaret	E		Oromia	East Shewa	Adama
Shashemene	E	U	Oromia	West Arsi	Shashemene
Sheno		U	Oromia	North Shewa	Kimbibit
Ziway	U	U	Oromia	East Shewa	Batu

U = Unexposed village or town. E = Exposed village or town. Southern Nations, Nationalities and People’s Region (SNNPR)
