# Supplementary material for: Owner reported diseases of working equids in central Ethiopia
Source: Equine Vet J. 2016 Oct 13;49(4):501–6. doi: 10.1111/evj.12633 (PMC5484383; doi:10.1111/evj.12633)
Supplement: Supplementary file 4 — Supplementary Item 4. Data on the population of horses and donkeys in each of the study regions. [file EVJ-49-501-s004.pdf]

Equine Veterinary Journal

Supplementary Item 4: Data on the population of horses and donkeys in each of the study regions (Federal Democratic Republic of Ethiopia Central Statistical Agency: Agricultural Survey 2013/14. Statistical Bulletin 573. August 2014).

	**Amhara n (%)**	**Oromia n (%)**	**SNNPR n (%)**	**Ethiopia Total**
**Horse**	404,814 (20.6)	1,186,437 (60.4)	366,008 (18.6)	1,963,010
**Donkey**	2,428,758 (34.9)	2,898,282 (41.7)	571,191 (8.2)	6,953,077

Southern Nations, Nationalities and People's Region (SNNPR)
