# Supplementary material for: Owner reported diseases of working equids in central Ethiopia
Source: Equine Vet J. 2016 Oct 13;49(4):501–6. doi: 10.1111/evj.12633 (PMC5484383; doi:10.1111/evj.12633)
Supplement: Supplementary file 5 — Supplementary Item 5. Semi‐structured interview questions and participatory methodologies used in the PSA. [file EVJ-49-501-s005.pdf]

Equine Veterinary Journal

Supplementary Item 5: Semi-structured interview questions and participatory methodologies used in the PSA.

Semi-structured interview questions	Methodology
What are the common diseases and health concerns that affect your horses and donkeys?	Open discussion and listing
How common are these conditions?	Ranking
How long do these conditions affect your horse, mules or donkeys ability to work?	Matrices[#] (disease volunteered against time period not able to perform work)
Do these problems affect your job, income and lifestyle?	Open discussion
What are the clinical signs associated with these diseases?	Open discussion

[#]Options in matrices (Never out of work, out of work for up to 1 day, out of work for up to 1 week, out of work for up to 1 month, out of work for greater than 1 month and permanently out of work).
