# Supplementary material for: Owner reported diseases of working equids in central Ethiopia
Source: Equine Vet J. 2016 Oct 13;49(4):501–6. doi: 10.1111/evj.12633 (PMC5484383; doi:10.1111/evj.12633)
Supplement: Supplementary file 6 — Supplementary Item 6. Complete list of all disease and health problems volunteered by 32 groups of horse‐ and donkey‐owners at 16 sites in Central Ethiopia. [file EVJ-49-501-s006.pdf]

Equine Veterinary Journal

Supplementary Item 6: Complete list of all disease and health problems volunteered by 32 groups of horse and donkey owners at 16 sites in Central Ethiopia.

Disease and Health Problems	Groups of horse owners that volunteered the disease (n^{max} = 16)	Groups of donkey owners that volunteered the disease (n^{max} = 16)	Disease and Health Problems	Groups of horse owners that volunteered the disease (n^{max} = 16)	Groups of donkey owners that volunteered the disease (n^{max} = 16)
Musculoskeletal	10	2	Nasal discharge	4	12
EZL	14	0	Diarrhoea	1	0
Coughing	10	11	Swelling above eye	2	0
Foot abscess	5	1	Swelling on neck	1	0
Colic	11	5	Swollen leg/lameness	4	0
Corneal opacity	1	0	Joint swelling	1	0
Parasites	5	5	Shivering	1	0
Wound on upper lip	6	0	Thin	1	0
Rubbing	1	1	Abdominal swelling	1	0
No urination	3	4	Rabies	0	5
Bloating	2	5	Weight loss	0	3
Circling	1	0	Hoof problem	0	3
Hair loss	1	1	Food poisoning	0	1
Knee Swelling	2	0	Limb abnormality	0	1
Wound	3	9	Drying of the back	0	1
Sarcoids	0	10	Leech	0	1
Anthrax	2	1	Abortion	0	1
Sudden Death	0	1	Eye infection	0	1
Day Disease	3	4	Swelling of eye	0	1
Mouth lesion	3	0	Bloody urine	0	1

EZL = Epizootic Lymphangitis, Day Disease = Unknown disease causing death within one day (clinical signs suggest possible aetiologies, particularly African Horses Sickness Virus), Musculoskeletal = musculoskeletal syndrome (with the local name “Bird” in horses, clinical signs suggest possible disease pathologies including equine exertional rhabdomyolysis), Parasites = internal parasites (described by owners as worms).
