# Supplementary material for: Owner reported diseases of working equids in central Ethiopia
Source: Equine Vet J. 2016 Oct 13;49(4):501–6. doi: 10.1111/evj.12633 (PMC5484383; doi:10.1111/evj.12633)
Supplement: Supplementary file 7 — Supplementary Item 7. Thematically coded clinical signs attributed by donkey‐owners to volunteered disease problems. [file EVJ-49-501-s007.pdf]

Equine Veterinary Journal

**Supplementary Item 7:** Thematically coded clinical signs attributed by donkey owners to volunteered disease problems.

| DONKEY OWNERS | | |
|---|---|---|
| **Problem (n = number of groups who volunteered this problem)** | **Thematic description** | **Number of groups that volunteered thematic description** |
| Nasal Discharge (n = 12) | Fluid/discharge from nose | 12 |
| | Head down | 4 |
| | Hole in throat [a] | 3 |
| | Not eating/drinking/breathing correctly | 2 |
| | Coughing | 2 |
| | Dead [b] | 4 |
| Coughing (n = 11) | Coughing | 11 |
| | Swelling under abdomen | 2 |
| | Fluid from nose | 4 |
| | Death | 2 |
| | Occurs at night time | 2 |
| Wound (n = 9) | Wound | 9 |
| | Awareness of wound locations | 9 |
| | Owner problem | 4 |
| | Sarcoid | 2 |
| Sarcoid (n = 10) | Location of sarcoids | 10 |
| | Swelling first | 4 |
| | Bleeding | 2 |
| | Thin skin | 2 |
| Bloating (n = 5) | Bloating | 5 |
| | No urination | 2 |
| | Loss of eating | 3 |
| | Reverse/not walking | 2 |

[a] Hole in throat likely to be burst abscess. [b] Owners described this disease problem presenting as death in their animals on certain occasions.
