# Supplementary material for: Owner reported diseases of working equids in central Ethiopia
Source: Equine Vet J. 2016 Oct 13;49(4):501–6. doi: 10.1111/evj.12633 (PMC5484383; doi:10.1111/evj.12633)
Supplement: Supplementary file 8 — Supplementary Item 8. Thematically coded clinical signs attributed by horse‐owners to volunteered disease problems. [file EVJ-49-501-s008.pdf]

Equine Veterinary Journal

Supplementary Item 8: Thematically coded clinical signs attributed by horse owners to volunteered disease problems.

HORSE OWNERS		
**Problem (n = number of groups who volunteered this problem)**	**Thematic description**	**Number of groups that volunteered thematic description**
EZL (n = 14)	Swelling and wound	13
	Lesions on body	12
	Disease spread	11
	Limb	8
	Blood vessels	4
	Disease severity	2
	Transmission	1
Musculoskeletal (n = 10)	Musculoskeletal change	9
	Locomotion	8
	Food and water	3
	Sweating	3
	Weight loss	1
Colic (n = 11)	Rolling	10
	Restlessness	8
	No urination	5
	Work associated	4
	Bloating	3
Coughing (n = 10)	Coughing	10
	Loss of appetite/thinner	5
	During/after work cough	3
	Foam and fluid	2
	Cough after eating	2
Lip wound (n = 6)	Swelling/wound on upper lip	6
	Reduced water and feed intake	6
